# Histological Confirmation of Myelinated Neural Filaments Within the Tip of the Neurotrophic Electrode After a Decade of Neural Recordings

**DOI:** 10.3389/fnhum.2020.00111

**Published:** 2020-04-21

**Authors:** Marla Gearing, Philip Kennedy

**Affiliations:** ^1^Laboratory Medicine and Neurology, Department of Pathology, Emory University School of Medicine, Atlanta, GA, United States; ^2^Neural Signals Inc., Duluth, GA, United States

**Keywords:** neurotrophic electrode, brainstem stroke, locked-in syndrome, single unit recordings, neurafilaments

## Abstract

**Aim:**

Electrodes that provide brain to machine or computer interfacing must survive the lifetime of the person to be considered an acceptable prosthetic. The electrodes may be external such as with electroencephalographic (EEG), internal extracortical such as electrocorticographic (ECoG) or intracortical.

**Methods:**

Most intracortical electrodes are placed close to the neuropil being recorded and do not survive years of recording. However, the Neurotrophic Electrode is placed within the cortex and the neuropil grows inside and through the hollow tip of the electrode and is thus trapped inside. Highly flexible coiled lead wires minimize the strain on the electrode tip. Histological analysis includes immunohistochemical detection of neurofilaments and the absence of gliosis.

**Results:**

This configuration led to a decade long recording in this locked-in person. At year nine, the neural activity underwent conditioning experiments indicating that the neural activity was functional and not noise. This paper presents data on the histological analysis of the tissue inside the electrode tip after 13 years of implantation.

**Conclusion:**

This paper is a singular example of histological analysis after a decade of recording. The histological analysis laid out herein is strong evidence that the brain can grow neurites into the electrode tip and record for a decade. This is profoundly important in the field of brain to machine or computer interfacing by implying that long term electrodes should incorporate some means of growing the neuropil into the electrode rather than placing the electrode into the neuropil.

## Introduction

Much effort and expenditure has gone into developing an electrode that will record neural signals for the lifetime of the paralyzed individual which is at least 50 years in a 20 year old injured person. Neural signals consist of low resolution electroencephalographic (EEG) signals, electrocorticographic (ECoG) signals, or multiunit or single units recorded from within the cortex. Electrodes are generally approximated to the neural source but over time are rejected by the gliosis that develops at the interface, eventually losing any useful signal. EEG electrodes with multiple recording surfaces can be safely replaced with each use, but the signals, though they are useful for low resolution tasks, have insufficiently high resolution for controlling smooth movements or producing conversational speech (Ang et al., 2015; Bundy et al., 2017; Monge-Pereira et al., 2017; Edlinger et al., 2018). ECoG signals are invasive and use the frequency domain recorded from groups of neurons and axons (Yanagisawa et al., 2011; Homer et al., 2013; Ikeda et al., 2014; Kanas et al., 2014; Martin et al., 2014, 2018; Mugler et al., 2014; Hotson et al., 2016; Jiang et al., 2016; Ramsey et al., 2017; Ibayashi et al., 2018). Future long term studies are likely to demonstrate gliosis developing at the ECoG/cortex interface. Intracortical recordings with the Blackrock array have resulted in recordings for several years, but published results indicate 85% loss of signal over 3 years (Downey et al., 2018). These results cannot justify its use as a lifetime recording electrode. A different approach is taken with the neurotrophic electrode (NE). Instead of approximating the electrode to the neurons, it induces the neurons to grow processes into the hollow tip using trophic factors as attractants (Kennedy, 1998). The neuropil grows through the hollow glass tip and becomes fixed to outside neuropil at both ends (Kennedy et al., 1992b). In addition, the tip is fixed to the end of a highly flexible coiled gold wire so that even macro movements of the brain that occur during running and jumping do not result in damage to the configuration as seen with monkey behavior (Kennedy and Bakay, 1998). The duration of recording is as long as 15 months in awake behaving monkeys (Kennedy and Bakay, 1997), 16 months in rats (Kennedy, 1998), short time in some humans (Kennedy et al., 1992a), and 4 years in two humans and 10 years in one human (subject 5) (Guenther et al., 2009; Brumberg et al., 2010, 2011; Kennedy et al., 2018) whose histology is reported here.

## Materials and Methods

### Ethical Approval

This study was approved by the human investigation committee of Neural Signals Inc., Gwinnett Medical Center. Approval was also obtained from the Food and Drug Administration (IDE#:G960032).

### The Subject

Subject 5 suffered a trauma-induced brainstem stroke at age 16. Following rehabilitation he remained locked-in and mute. He could move his eyes to indicate “yes” or “no,” and had difficulty in controlling facial movements, with only a very slow blink. He had monotonic vocalization and diplopia with abnormal foveation. He attended visually to targets using the peripheral aspect of his retina. He had intact hearing and so he enjoyed listening to music. He could respond to probing questions with eye blinks, strongly suggesting his intelligence was intact even though formal neuropsychological testing was not possible. Structural MRI of his brain indicated brainstem stroke but no hemispheric damage, and functional MRI indicated normal activation of his speech cortex during a naming task. He was implanted with the NE in 2004 at age 22 years. Functional recordings were performed at year nine (Kennedy et al., 2018) and final recordings at year 10 (2014). Beyond that point, he was too ill to participate in studies due to extension of his stroke to his autonomic brainstem centers resulting in a drop in blood pressure with syncope even when merely elevating his head. He died at age 34, thirteen years after electrode implantation. The electrode and surrounding tissue were recovered and histology was performed. His father gave permission for the removal of the electrode and surrounding cortex.

### The Electrode

**FIGURE 1:**
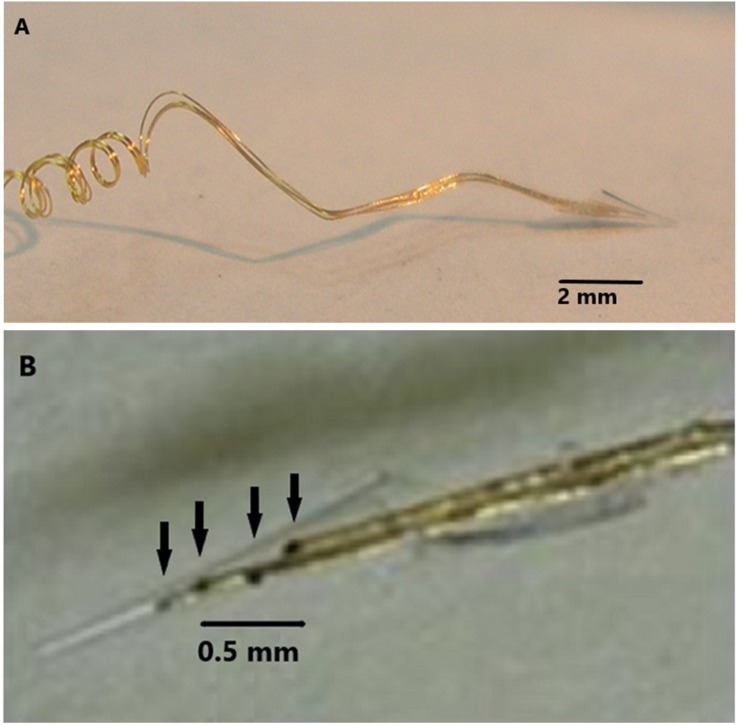
**(A)** The electrode wires (coils on left) are wound on a glass pipette and then shaped so that the electrode cannot enter too deeply into the cortex. **(B)** Demonstrates four wires with dark contact surfaces inside the glass cone tip indicated by arrows.

The NE was hand assembled under a microscope. 2 mil Teflon insulated 99.9% gold wires were wound around a pipette to the desired length, usually 1 to 3 cm as shown in Figure 1A. A glass pipette was pulled and the tip was cut off so that the diameter of the tip was 50 microns at the deep end and 200–300 microns at the wide upper end as shown in Figure 1B. The cone shaped tip was 1.5 to 2 mm in length. In subject 5’s case, three gold wires were inserted into the tip and glued inside with methyl methacrylate. The inserted ends of the wires were offset so that only one wire approached the deep end and remained about 500 microns from that end. The upper end of the coiled wires were soldered to a connector and insulated with medical grade acrylic. Further details are found here (Bartels et al., 2008). An example of the final assembly is shown in Figure 1.

### Implantable Electronics

**FIGURE 2:**
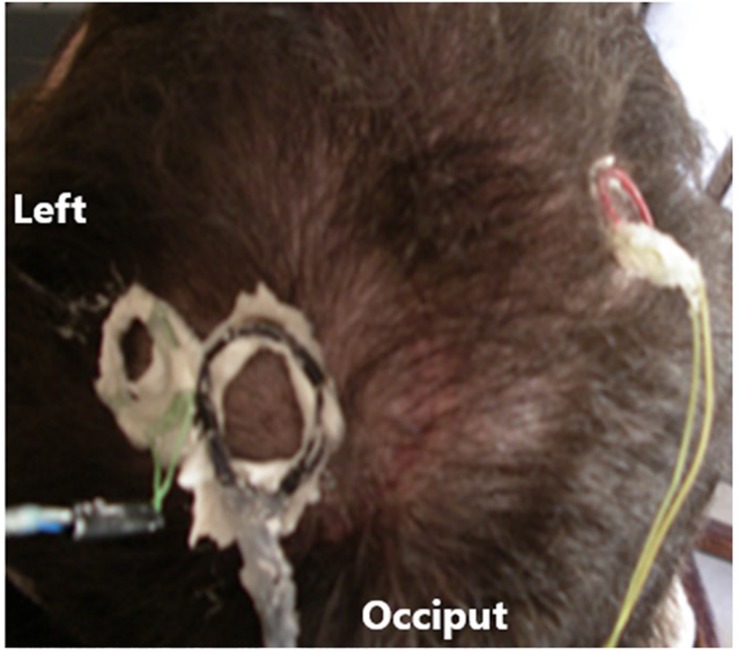
Head of subject 5 with attached coils.

A duel channel implantable electronic device was implanted in subject 5 under the scalp above the skull with the electrode implanted in the speech cortex to record the neural signals and transmit them using a frequency modulated carrier (FM). A power induction coil was placed over an implanted receiving coil to power up the device and turn it on. Amplifiers (typical gain × 800, band pass 5 to 5,000 Hz), connected to the electrode wires and secured to the skull and the electrode with acrylic cement. The amplified neural signals were transmitted through the scalp using an FM transmitter operating in the 42 ± 8 MHz range for external processing. During the recording sessions, the power induction coil and the FM receiving coils were secured to the scalp, using EEG paste (Grass Technologies) to provide stability as shown in Figure 2. The demodulated signals (WiNRADiO Communications, Adelaide, SA, Australia) were passed through CWE amplifiers (Ardmore, PA; 20× gain, band pass-filtered 3–10 kHz). The analog data were archived on a DDS tape recorder (Cygnus Technology, Inc., United States) for offline analysis. After A/D conversion, the digital filters were set at 300 Hz to 6 KHz for single units and 1 Hz to 6 KHz for continuous recordings. Trauma to the electronics due to handling of the head during hygiene led to replacement of the units on three occasions. The electrodes have never needed replacement. The electronic units were protected by Elvax internally and Silastic externally for insulation and trauma protection, respectively. The recording electronics were totally passive, contain no batteries, and were not used for stimulation so there was so danger of electrical discharge.

### Recorded Data Analysis

**FIGURE 3:**
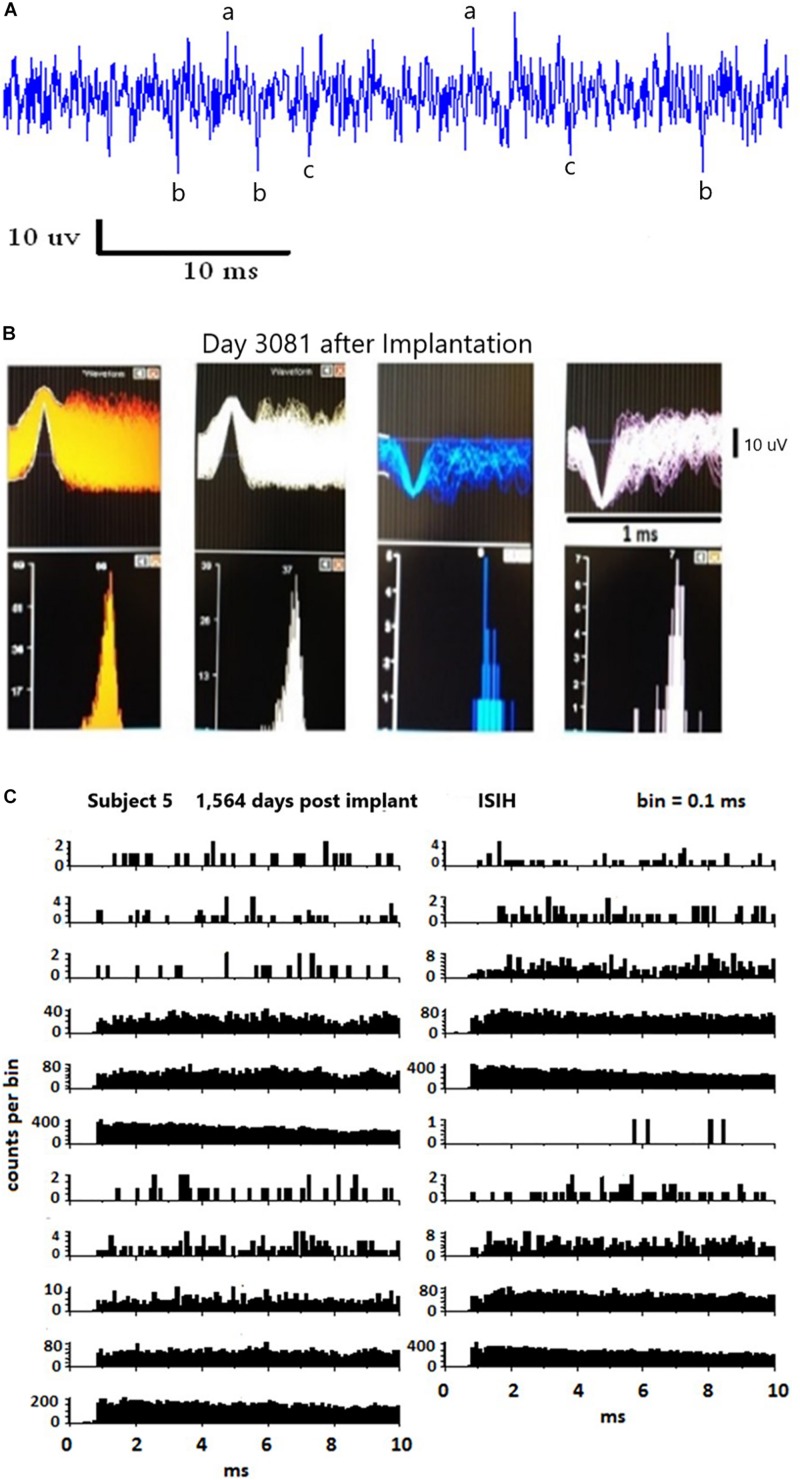
**(A)** Illustrates a 40 ms continuous data stream. Note the low amplitude of the data. The potential single units are labeled (a,b,c). Discriminating these potential signals from noise using auto-correlograms is illustrated in **(B)**. Note the single peaks in the autocorrelations. Inter-spike interval histograms five years after implantation are illustrated in **(C)**. These data are valid only for fast firing units, as shown.

Neural signal amplitudes ranged from 12 to 80 μVpp. System noise was 10 uVs. Spike events were first detected by positive and negative voltage threshold crossings at ±10 μV. A 32-point (∼1 ms) waveform was taken around the threshold crossing, peak-aligned to the eighth sample and saved for further analysis. Total acquisition system setup time varied between 10 and 20 min. Thresholded spike event waveforms were sorted first manually offline with the Neuralynx, Inc. SpikeSort3D program (Bozeman, Montana), utilizing a standard manual convex-hull cluster-cutting algorithm according to five features: peak/valley amplitude, height (peak-to-peak amplitude), and energy (area under the spike) and amplitude at the spike alignment point. The eighth sample amplitude was used as the primary separation feature. It ideally corresponds to the maximum peak or valley amplitude. Spike timestamps were recorded and stored according to the alignment point of the clustered waveform (eighth). The final feature space parameters derived from this off-line analysis were saved for use over all recording sessions in this study. Only when the electronic implants were changed out due to traumatic damage were the parameters altered. Figure 3 illustrates in *Panel A* an example of continuous data where similar single units are labeled. *Panel B* illustrates four single units on a 1 ms time base and small amplitude. To discriminate these units from noise, auto-correlograms show only single peaks, and strongly suggesting single units. Further analysis using inter-spike interval histograms (*Panel C*) illustrates the approximately 1 ms gap between the firings indicating that these are individual units. These data are valid when the firing rate is sufficiently high, which is not the case for the slow firing units as shown.

### Histological Methods

**FIGURE 4:**
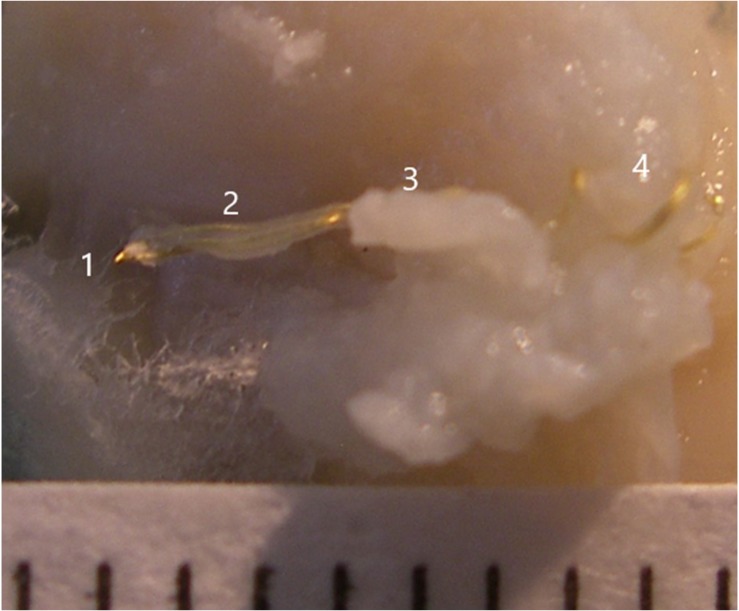
Illustration of the electrode wires and tissue that was inside the glass cone. The numbers 1, 2, 3, and 4 indicate positions 1, 2, 3, and 4, respectively.

One day after dying, the electrode and surrounding cortical tissue were removed. Under the microscope, the electrode tissue was dissected and the three wires were cut at about number 3 position in Figure 4. They were then removed one at a time with a forceps holding one end of the tissue at position 3 and a second forceps holding the wire at position 1 and drawing it out. This avoided damage to the tissue. Figure 4 illustrates the electrode wires (position 1) after the glass cone was removed. Position 2 demonstrates the neural tissue inside the cone with the wires visible through it. Position 3 demonstrates the tissue outside the glass cone after removal. Position 4 is adjacent to the coiled wires.

Tissue was immersion fixed in 4% paraformaldehyde for several weeks at room temperature, then embedded in paraffin and serially sectioned at 10 microns. Every fifth section was stained with hematoxylin and eosin (H&E). Additional sections were stained with myelin stain (Luxol fast blue-periodic acid Schiff; LFB-PAS); positive control slides were included to confirm successful staining. Other sections were immunohistochemically labeled with antibodies to neurofilament (Dako/Agilent, clone 2F11, 1:100) and GFAP (Dako/Agilent, rabbit polyclonal Z0034, 1:5000) on a ThermoFisher Scientific autostainer using the ThermoScientific UltraVision LP detection system per manufacturer’s recommendations. Specifically, sections were exposed to 3% hydrogen peroxide in methanol for 5 min, incubated with UltraVision block for 10 min, exposed to primary antibody diluted in Tris buffer for 1 h, and incubated with Primary Antibody Enhancer for 10 min followed by horseradish peroxidase (HRP) Polymer for 15 min. Sections were washed in buffer between each step, and all incubations were carried out at room temperature. 3,3′ diaminobenzidine (Immpact DAB, Vector Labs) was used for color development (approximately 5 min at room temperature), and sections were counterstained with hematoxylin. Positive controls and negative controls were included in each immunohistochemical staining run.

## Results

### Recording

**FIGURE 5:**
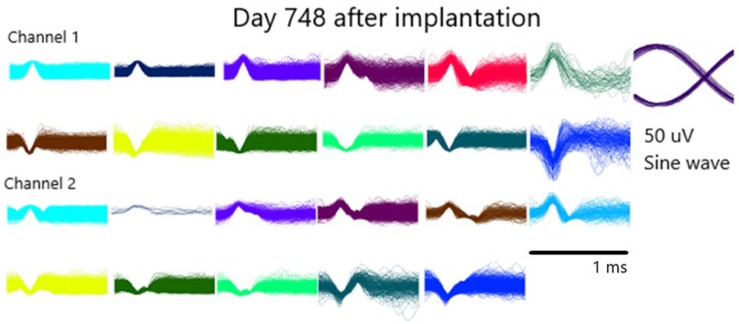
Illustration of the waveforms of single units from both channels on day 748 after implantation.

Recorded data have been extensively published elsewhere demonstrating functionality of the pattern of firing of single units (Kennedy et al., 1992a, b, 2017, 2018; Kennedy, 1998, 2018; Kennedy and Bakay, 1997, 1998; Kennedy, 1998; Guenther et al., 2009; Brumberg et al., 2010, 2011). An example of single unit activities is provided in Figure 5 from day 748 after implantation. These units illustrate the positive and negative deflections from both data channels. Also shown is the sinewave of 50 uVs that was used as a calibration signal by being recorded along with the data.

### Histology

**FIGURE 6:**
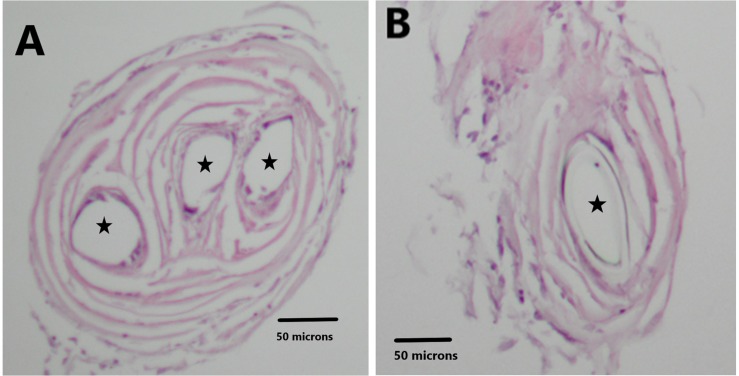
H&E stain illustrates the holes in the tissue related to the recording wires. Panel **(A)** shows three circular openings (black stars). Panel **(B)** shows one circular opening (black star).

Ten-micron cross sections of the H&E stained tissue are illustrated in Figure 6. *Panel A* demonstrates the three circular openings (black stars) in the neural tissue due to the three recording wires that were lodged in it. Panel B shows one opening (black star). *Panel B* is sampled distal to *Panel A*. These data confirm that the recording wire tips were separated from each other in the longitudinal direction inside the cone. The distance between the end of the three wire section and the end of the one wire section is 210 microns. One wire was used as a reference for the other wires thus creating channels 1 and 2 (see section “Materials and Methods”). Thus it is likely that the data at these recording points ought to be different from each other.

#### Neurofilaments

**FIGURE 7:**
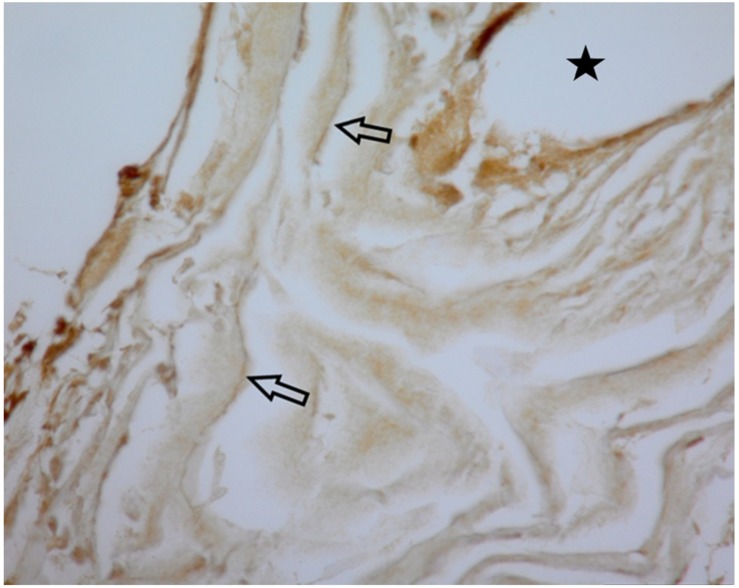
This Panel illustrate the stained neurofilaments indicated by arrows. The black star indicates the opening for the electrode wire. Magnification is ×40.

The magnified sections (high-power images (×40) in Figure 7) illustrate the neurofilament-containing processes that wind upward in a near vertical direction. There are many neurofilaments, which are likely axons, in each section.

#### Myelination

**FIGURE 8:**
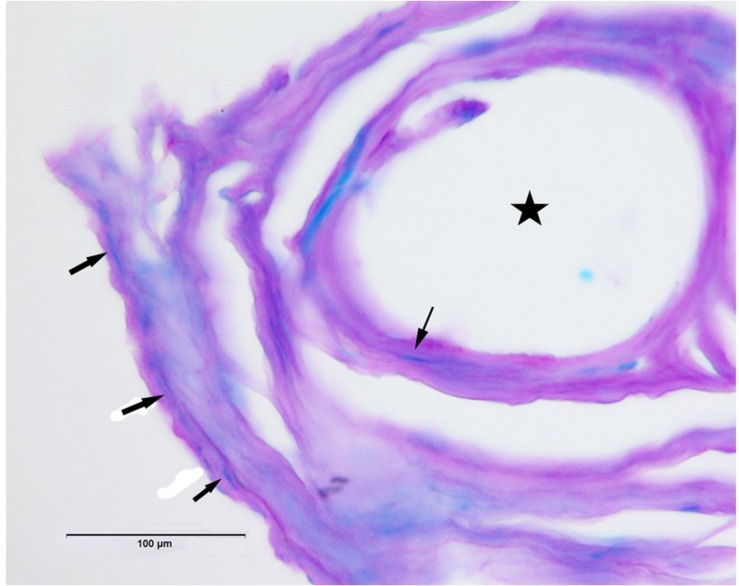
Luxol fast blue-periodic acid Schiff (LFB-PAS) stain shows examples of myelinated axons in blue (arrows). Star indicates hole in tissue where once the electrode wire resided.

Luxol fast blue-periodic acid Schiff stained sections showed areas with blue staining, indicative of myelinated axons as shown in Figure 8. The gold recording wires record multiple units from these neuronal processes which are then separated as described in section “Materials and Methods.”

#### Gliosis

**FIGURE 9:**
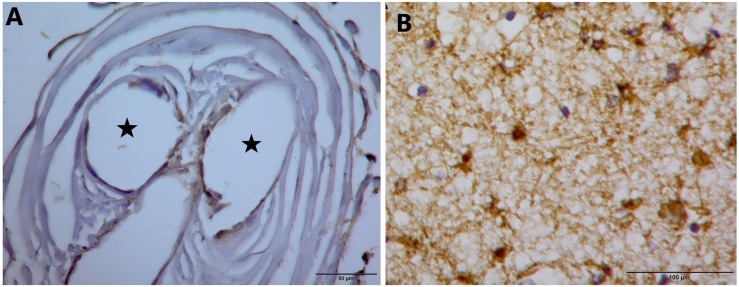
**(A)** GFAP stain on tissue from inside the electrode tip does not demonstrate gliosis. Black stars indicate holes in tissue where once the electrode wires resided. **(B)** GFAP stain that demonstrates glial cells in a separate control piece of tissue.

Figure 9 illustrates, using GFAP immunohistochemistry, the lack of evidence for gliosis in *Panel A* for subject 5 and the presence of glial cells in *Panel B* as a positive control.

## Discussion

This is the first report of histological confirmation of *decade long* recorded neural activity. These histological data demonstrate the persistence of neurofilament-containing neuronal processes recorded for a decade, and their persistence for three more years until subject 5 died. These data confirm the original data on the rat and monkey histology that showed electron microscopic evidence of myelinated axons, axo-dendritic synapses, blood vessels, no neurons and no gliosis or microglia, from 3 weeks to 16 months after implantation (Kennedy et al., 1992b; Kennedy, 1998). There were other human histological reports which were limited by autopsy technical problems but published nevertheless [subject 1, Kennedy and Bakay, 1998], refusal to allow retrieval of the histology [subject 2], uselessness of the neuropil in an end stage mitochondrial myopathy person [subject 3], and one other whom the histology is unavailable [subject 4]. The sixth implanted subject is this author who still has the electrode tips lodged in his speech motor cortex which will be retrieved upon his death.

The present paper is profoundly important to the field of brain computer interfacing. It confirms that the survival of neurofilaments is allied with continuing recording of neural activity for as long as a decade until subject 5 became too ill to participate (Kennedy et al., 2018). Conditioning studies were performed at year nine demonstrating that the signals were not artifactual noise (Kennedy et al., 2018). The basis of the electrode is that the neuropil is encouraged to grow into and through the hollow tip which therefore remains securely in the neuropil alongside the recording wires. Coiling of the lead wires outside the cortex reduces the strain on the implanted tip so that the structure remains intact despite slight movements of the brain during breathing, and more active movements during running and jumping as in monkeys (Kennedy et al., 1992a; Kennedy and Bakay, 1997). These results should drive workers in the field to rethink their choice of electrode when longevity is paramount, as it is with neural prostheses for restoring speech in the locked-in, restoring smooth movements in quadriplegics, controlling robotic arms and so on. All other electrodes are based on the premise of trying to maintain stability after insertion of the tip into the neuropil. It just does not happen over time: The Blackrock array loses 85% of its signals over 3 years (Downey et al., 2018). Nevertheless, these tine type electrodes are immensely useful in the short term whereas the NE requires 3 months for growth of neurites into the tip so is only useful after this short term period. The ingrowth of neuropil is induced by trophic factors (Kennedy et al., 1992a, b, 2017, 2018; Kennedy, 1998; Kennedy and Bakay, 1997, 1998; Kennedy, 1998; Guenther et al., 2009; Brumberg et al., 2010, 2011).

**FIGURE 10:**
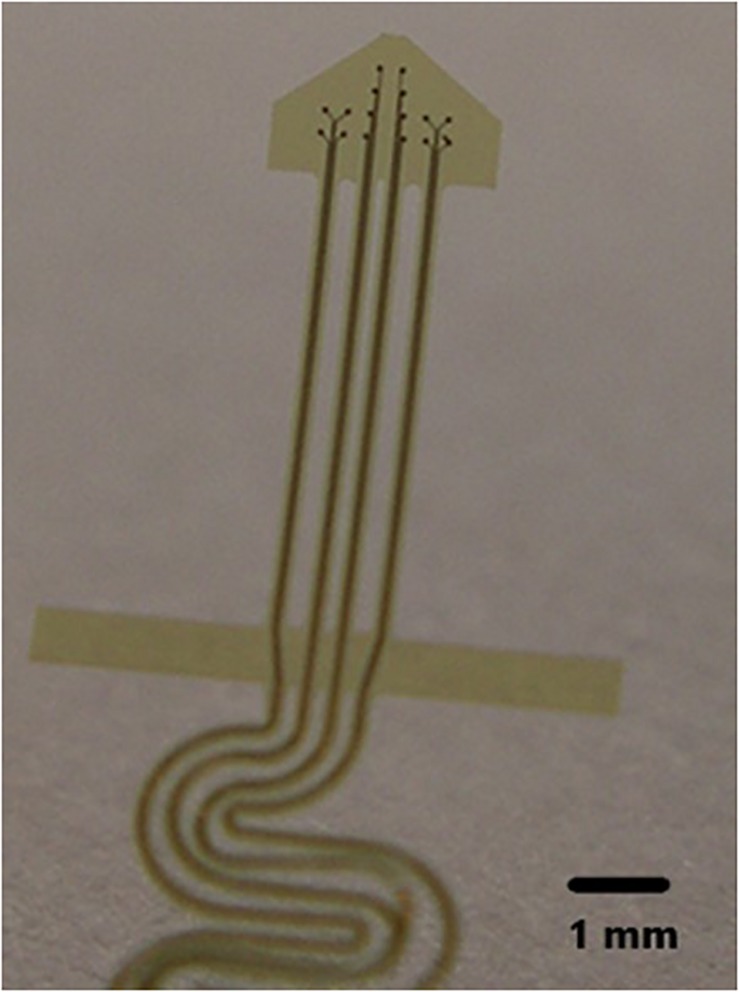
Illustration of a polyimide electrode.

Future improvement of the NE consists of increasing the number of recording surfaces within the hollow tip while at the same time maintaining the electrically insulating properties of the glass tip. Microfabrication on a polyimide base with increasing numbers of recording surfaces has been attempted in the past [personal observations] as can be seen in Figure 10. These were unsuccessful due to our inability (a) to turn them into a cone shape or (b) wrap them and place them inside the glass cone. Other variants on this effort are underway.

Electrocorticographic electrodes are being studied for analysis of speech and possibly a speech prosthetic (Ramsey et al., 2017; Anumanchipalli et al., 2019). However, they have not been tested in the long term, with one exception (Ramsey et al., 2017), because they are usually in place for weeks to assess seizure foci for surgical removal of the foci. Ramsey et al. (2017) have implanted ECOG electrodes for longer term usage, they will likely suffer the same fate as tine type electrodes, namely, gliosis will develop over time between the electrodes and the underlying cortex. However, a trial of longevity is worthwhile because they do not record from individual neurons or axons but rather from foci of many neurons or axons, so that loss of signal amplitude may not be so critical because frequency is the key to the analysis. It will be interesting to see how those studies turn out. The difference in risk between intracortical recordings as described here and ECoG cortical surface recordings is insertion to a depth of 6 mm for the intracortical electrodes. So the risks of invasion are approximately equal for both systems with a tilt toward more danger for the intracortical electrodes due to possible intracortical hemorrhage or infection. Infections and hemorrhage with the cortical surface based ECoG electrodes are also possible.

## Conclusion

These data demonstrate that persistent human recordings over a decade is allied with histological confirmation of underlying neurofilament growth into the electrode tip with no gliosis. It is reasonable to expect further useful survival of the electrode past the 10 years milestone.

## Data Availability Statement

The datasets generated for this study are available on request to the corresponding author.

## Ethics Statement

This study was reviewed and approved by Neural Signals Inc., IRB. The patients legal guardian provided written informed consent for both participation and publication of the report and the patient provided assent.

## Author Contributions

MG performed the histological analysis after PK performed the recording over many years and removed the electrode from the subject’s brain on his demise.

## Conflict of Interest

PK was employed by the company Neural Signals Inc. He owns 98% of the common stock in the company. The remaining author declares that the research was conducted in the absence of any commercial or financial relationships that could be construed as a potential conflict of interest.
